# An Adult Case of Diabetes With High Levels of GAD Antibodies Without Insulin Deficiency for More Than 10 Years

**DOI:** 10.1155/crie/9712659

**Published:** 2025-02-27

**Authors:** Hideyuki Takeuchi, Naoyuki Iso-O, Atsuko Takai, Takashi Mikamo, Kozue Nagumo, Masumi Hara

**Affiliations:** Department of Internal Medicine, Teikyo University Hospital, Mizonokuchi, Kawasaki, Kanagawa, Japan

## Abstract

Latent autoimmune diabetes in adults (LADA) or slowly progressive insulin-dependent diabetes mellitus (SPIDDM) is a form of autoimmune diabetes characterized by autoimmune destruction of pancreatic beta cells, leading to deficient insulin secretion. Here, we report a case of diabetes and obesity in a 56-year-old woman. She was diagnosed with severe obesity, impaired glucose tolerance (IGT), and a positive antiglutamic acid decarboxylase antibody (GADA) test result at the age of 39 years. She developed diabetes 7 years later, meeting the diagnostic criteria for SPIDDM (probable). Despite high GADA levels, her endogenous insulin secretion has been preserved for over a decade. GADA has been regarded as a marker of autoimmune destruction of pancreatic beta cells, and high levels of GADA are considered a risk factor for future insulin deficiency. However, the role of GADA in its pathogenesis remains unclear. GADA is not a specific indicator of autoimmune diabetes, as it is also positive for autoimmune diseases such as autoimmune thyroid diseases (AITDs) and stiff-person syndrome. Therefore, a positive GADA test alone is not sufficient to predict insulin deficiency in an individual case, even if the titer is high. In the early stages, autoimmune diabetes presents clinical features similar to those of type 2 diabetes, particularly obesity. Although insulin therapy is often started early in the treatment of autoimmune diabetes, as the consensus statement indicates, when endogenous insulin secretion is preserved and the risk of insulin deficiency is low, efforts should be made to prevent body weight gain and the development of cardiovascular diseases (CVD) by following treatment guidelines for type 2 diabetes, with the exception of the use of sulfonylurea agents.

## 1. Introduction

Latent autoimmune diabetes in adults (LADA) is one of the adult-onset autoimmune diabetes, characterized by autoimmune destruction of pancreatic beta cells, leading to deficient insulin secretion. A pathological condition similar to LADA is called slowly progressive insulin-dependent diabetes mellitus (SPIDDM) [1, 2]. SPIDDM differs from fulminant [3] and acute-onset [4] type 1 diabetes because it causes slow pancreatic beta cell destruction and has early clinical features similar to those of type 2 diabetes. Autoantibodies such as antiglutamic acid decarboxylase antibody (GADA), anti-insulinoma-associated antigen-2 (IA-2) antibody, anti-insulin autoantibody, and anti-zinc transporter protein 8 (ZnT8) antibody are often associated with the autoimmune destruction of pancreatic beta cells. Several risk factors for insulin deficiency in patients with autoimmune diabetes have been reported [5–7]. Among them, high GADA levels, especially as measured by enzyme-linked immunosorbent assay (ELISA), have been reported to be a risk factor for insulin deficiency.

It has been reported that the early introduction of insulin therapy for LADA and SPIDDM may preserve pancreatic beta cell function [8, 9]. However, some patients with autoimmune diabetes do not progress to insulin deficiency over long periods [10, 11]. Many of these patients are obese and share clinical features with those with type 2 diabetes. Early introduction of insulin therapy in these patients may lead to weight gain, worsen long-term glycemic management, and increase the incidence of atherosclerotic cardiovascular disease (ASCVD). Although there are no treatment guidelines for LADA or SPIDDM, the consensus statement by an international expert panel [12] and the report by the Japan Diabetes Society that presents the diagnostic criteria for SPIDDM [2] recommend that, in cases where endogenous insulin secretion is maintained, a treatment plan should be formulated in accordance with the guidelines for type 2 diabetes, with the exception of the use of sulfonylurea agents.

Here, we report a case that met the diagnostic criteria for SPIDDM (probable) with persistently high GADA levels measured by ELISA, although the patient had obese type 2 diabetes-like clinical features with no insulin deficiency for more than 10 years after being diagnosed with impaired glucose tolerance (IGT).

## 2. Case Presentation

**Figure 1:**
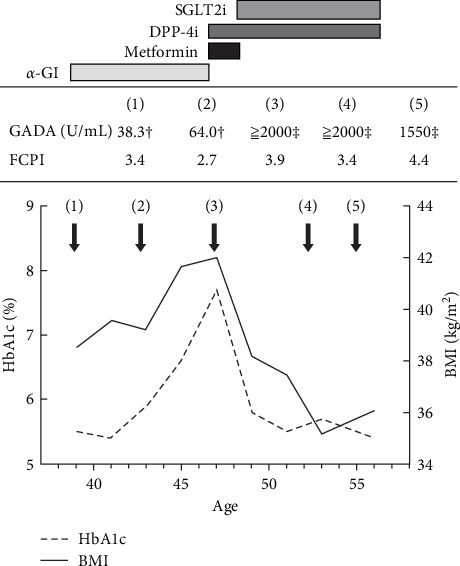
Clinical course changes in HbA1c, body weight, medications, GADA level, and fasting CPI, an indicator of endogenous insulin secretion, are shown. Values of GADA measured by RIA are indicated by † and those measured by ELISA by ‡. Fasting CPI (ng/mg) was calculated using the following formula: FCPR (ng/mL)/fasting blood glucose (mg/dL) × 100. α-GI, alpha-glucosidase inhibitors; DPP-4i, dipeptidyl peptidase-4 inhibitors; ELISA, enzyme-linked immunosorbent assay; FCPI, fasting C-peptide index; FCPR, fasting C-peptide immunoreactivity; GADA, antiglutamic acid decarboxylase antibody; RIA, radioimmunoassay; SGLT2i, SGLT2 inhibitors.

The patient was a 56-year-old Japanese woman with severe obesity and a history of Graves' disease. At the age of 39, she came to our hospital with suspicion of IGT. At the time of her first visit, her BMI was 38.5 kg/m^2^, and a 75 g oral glucose tolerance test (OGTT) indicated IGT and insulin resistance (Table 1). No dyslipidemia or hypertension was observed. At the same time, the patient tested positive for GADA. The subsequent clinical course is shown in Figure 1. She had a history of autoimmune thyroid diseases (AITDs); she had Graves' disease in her twenties but was in remission at the time of her first visit and was positive for anti-TPO and antithyroglobulin antibodies. Her thyroid function had been within normal limits since her first visit. After several months of lifestyle modifications, the patient was started on an alpha-glucosidase inhibitor to prevent progression to diabetes mellitus. At the age of 46, she progressed from IGT to diabetes based on elevated fasting blood glucose and HbA1c levels. Her GADA levels remained extremely high (≥2000 U/mL in ELISA), but fasting C-peptide levels did not indicate a decrease in endogenous insulin secretion. Tests for other islet-associated autoantibodies, such as anti-IA-2, anti-ZnT8, and anti-insulin antibodies, were negative. Metformin was started in place of an alpha-glucosidase inhibitor, followed by the addition of a DPP-4 inhibitor. In 2016, the method of measuring GADA changed from radioimmunoassay (RIA) to ELISA in Japan. Since then, her GADA levels have remained high, above 2000 U/mL. At the age of 47 years, an SGLT2 inhibitor was started for the purpose of weight loss, and metformin was withdrawn because of good glycemic control. At the age of 56 years, she was obese with a BMI of 35 but maintained good glycemic management with an HbA1c level of ~6% on DPP-4 and SGLT2 inhibitors. Fasting blood CPR did not decrease, at ~4 ng/mL, and GADA values slightly decreased but remained high at 1300–1600 U/mL.

## 3. Discussion

LADA is defined as diabetes that develops in people aged 30 years or older, who have at least one islet cell-associated autoantibody, and who are insulin-independent for at least 6 months after diagnosis [13]. This case fits the definition because the patient developed diabetes at the age of 46 years and did not require insulin treatment for a long time, despite having high GADA levels. In addition, this case met the diagnostic criteria for “SPIDDM (probable)” set by the Japan Diabetes Society. Considering the patient's significant obesity and preserved insulin secretion, this case should rather be diagnosed as SPIDDM (probable) and should be followed up while assessing the degree of insulin secretion ability.

This case provides two implications for determining treatment strategies for autoimmune diabetes. First, a single positive GADA result, even if measured by ELISA and a high titer, is not sufficient to predict the risk of insulin deficiency in an individual case. Second, for people with autoimmune diabetes who are obese and exhibit the clinical features of type 2 diabetes, the prevention of body weight gain and development of cardiovascular diseases (CVDs) are important treatment goals. Periodic evaluation of endogenous insulin secretion is necessary, and basic pharmacotherapy should be performed in accordance with the treatment guidelines for type 2 diabetes, except for the use of sulfonylurea agents.

A single positive GADA, even at a high titer, is not sufficient to predict the risk of insulin deficiency in individual cases. The diagnosis of LADA and SPIDDM requires the presence of positive islet-specific autoantibodies, such as GADA, anti-IA-2 antibody, anti-insulin autoantibody, and anti-ZnT8 antibody. Although these autoantibodies are detected in association with autoimmune injury to pancreatic beta cells, the autoantibodies themselves may not be the direct cause of pancreatic beta cell injury but rather merely markers of the autoimmune response to pancreatic beta cells [14]. Furthermore, GADA is known to be positive in other autoimmune diseases such as AITD and stiff-person syndrome [15]. GADA is also known to be positive in some people with phenotypic type 2 diabetes and even in healthy individuals [16]. Therefore, GADA is not a specific marker of pancreatic beta cell injury.

In contrast, GADA is the most commonly used sensitive marker for diagnosing LADA and SPIDDM, and high GADA titers have been reported to be associated with a subsequent decrease in endogenous insulin secretion [5, 7, 17, 18]. In a Swedish population study using GADA 250 IU/mL as a cutoff and dividing the patients into high or low, the percentage of patients requiring insulin therapy at 6 months was 42.2% for the high-titer group compared with 3.7% for phenotypic type 2 diabetes [19]. In particular, the clinical utility of GADA has improved since the change of the assay system from RIA to ELISA [20]. However, in some cases, such as the present case, endogenous insulin secretion is not reduced for a long period of time [10]. According to the American Diabetes Association (ADA) stage classification of type 1 diabetes, presymptomatic type 1 diabetes (stage 1) is defined as having two or more type 1 diabetes-associated islet autoantibodies [21]. Similarly, in LADA and SPIDDM, the presence of multiple islet-associated autoantibodies may be helpful in assessing the risk of future insulin deficiency. For the report on GADA measured by RIA, there is also a report that patients who tested positive for multiple antibodies were at a higher risk of requiring insulin therapy than those who tested positive for GADA alone [22].

In addition to GADA, various other risk factors for insulin deficiency in patients with autoimmune diabetes have been reported (Table 2). Although the GADA level was extremely high in the present case, many other aspects suggested a low risk of insulin deficiency. No islet-associated autoantibodies other than GADA were detected, the BMI was high, and there was no evidence of decreased endogenous insulin secretion. This patient did not have any HLA genotypes reported to be susceptible to type 1 diabetes in the Japanese population but had DRB1*⁣*^*∗*^15:02 and DQB1*⁣*^*∗*^06:01, which are considered protective [5, 26]. Based on the above, the risk of developing insulin deficiency in this case was estimated to be low, even considering the high GADA level. Although this is only a case report, in patients who do not develop insulin deficiency even after a certain period after the diagnosis of autoimmune diabetes, as in the present case, HLA genotype analyses may be useful for risk assessment of insulin deficiency and subsequent treatment decisions. A positive anti-TPO antibody result has been reported to be a risk factor for insulin deficiency in LADA [24]. However, GADA is sometimes positive in AITD, although its relationship with the pathogenesis is not clear. Therefore, when GADA is the only positive islet cell-related autoantibody in patients with AITD, the possibility of a GADA-positive result other than islet-associated autoimmunity should be considered.

At present, there are no treatment guidelines for LADA or SPIDDM; however, a consensus statement by an international expert panel [12] and diagnostic criteria for SPIDDM [2] have been presented. In these reports, while endogenous insulin secretion was preserved and the risk of insulin deficiency was low, it is recommended that treatment be carried out in accordance with the guidelines for type 2 diabetes, except for the use of sulfonylurea agents. Early introduction of insulin therapy tends to be the treatment of choice after the diagnosis of autoimmune diabetes, as there are reports [9, 25] that early introduction of insulin therapy for LADA and SPIDDM has contributed to the preservation of endogenous insulin secretion compared with sulfonylurea. However, in obese patients, as in the present case, early insulin induction increases weight gain, thereby worsening long-term glycemic management and increasing the risk of ASCVD due to hyperinsulinemia. The risk of death in patients with LADA has been reported to be as high as that in patients with type 2 diabetes, and the CVD risk is particularly high when the GADA levels are high, as in this case [19].

The primary goals in the treatment of this patient were to prevent body weight gain and CVD. After being diagnosed with diabetes mellitus, the patient was started on metformin and SGLT2 inhibitors, followed by lifestyle modification guidance and good glycemic management. DPP-4 inhibitors were also administered because small clinical trials have suggested that they may retard insulin deficiency in patients with autoimmune diabetes [27–30]. Administration of GLP-1 or GLP-1/GIP might also be helpful if glycemic management worsens or if weight loss is difficult [31].

## 4. Conclusion

We report a case of obese diabetes that met the diagnostic criteria for SPIDDM (probable), with GADA levels remaining extremely high for over 10 years, but without a decline in endogenous insulin secretion. Even if GADA is high in a highly specific ELISA assay, a positive result for GADA alone is not sufficient to predict insulin deficiency in an individual case. Therefore, multiple islet-associated autoantibodies and endogenous insulin secretion should be evaluated. Obese patients with autoimmune diabetes and preserved endogenous insulin secretion should be treated according to the guidelines for type 2 diabetes with regular assessment of endogenous insulin secretion, except for the use of sulfonylurea agents.

## Figures and Tables

**Table 1:** Laboratory data.

At the first visit				At the time of diagnosis of diabetes						
		Normal range	Units			Normal range	Units			
FPG	113	70–110	mg/dL	FPG	144	70–110	mg/dL	HLA serotype		
HbA1c	5.5	4.6–6.2	%	HbA1c	6.6	4.6–6.2	%	A	A2	A24
GADA (RIA)	32.3	≦1.4	U/mL	GADA (ELISA)	≧2000	<5.0	U/mL	B	B39	B52
Insulin	33.6	2.2–12.4	μIU/mL	FCPR	3.86	0.8–2.5	ng/mL	DR	DR4	DR15
HOMA-IR	9.37	≦ 1.6	—	FCPI	2.7	—	—	—	—	—
HOMA-β	242	—	%	IA-2 Ab	<0.6	<0.6	U/mL	HLA genotype		
	—	—	—	Insulin Ab	<0.4	<0.4	U/mL	—	Allele 1	Allele 2
75 g OGTT				ZnT8 Ab	<10.0	<10.0	U/mL	A	02:06	24:02
Glucose (mg/dL)	—	—	—	FT4	1.2	0.90 – 1.70	ng/dL	B	39:04	52:01
0 min	113	—	mg/dL	FT3	3.1	2.30 – 4.00	—	DPB1	02:01	09:01
30 min	169	—	mg/dL	TSH	0.4	0.610 – 4.230	μIU/mL	DQB1	03:02	06:01
60 min	194	—	mg/dL	TRAb	<0.1	<2.0	IU/L	DQA1	01:03	03:01
120 min	125	—	mg/dL	TPO Ab	546	<16.0	IU/mL	DRB1	04:03	15:02
Insulin (μIU/mL)	—	—	—	Thyroglobulin Ab	244	<28.0	IU/mL	—	—	—
0 min	33.6	—	μIU/mL	—	—	—	—	—	—	—
30 min	116.8	—	μIU/mL	—	—	—	—	—	—	—
60 min	202.3	—	μIU/mL	—	—	—	—	—	—	—
120 min	205.4	—	μIU/mL	—	—	—	—	—	—	—

Abbreviations: 75 g OGTT, 75 g oral glucose tolerance test; ELISA, enzyme-linked immunosorbent assay; FCPI, fasting C-peptide index (FCPI = FCPR/FPG × 100); FCPR, fasting C-peptide immunoreactivity; FPG, fasting plasma glucose; GADA, antiglutamic acid decarboxylase antibody; HLA, human leukocyte antigen; IA-2 Ab, anti-insulinoma-associated antigen-2 antibody; insulin Ab, anti-insulin antibody; RIA, radioimmunoassay; TPO Ab, anti-TPO antibody; TRAb, anti-TSH receptor antibody; ZnT8 Ab, anti-zinc transporter protein 8 antibody.

**Table 2:** Risk factors for insulin deficiency in LADA.

Positive risk factors	The current case
High levels of GADA [5–7, 17, 18, 22]	Yes (>2000 U/mL by ELISA)
Multiple positive islet-related autoantibodies [23]	No
Low fasting C-peptide levels [5, 18]	No
High HbA1c at diagnosis [6]	No
Low BMI at diagnosis [6, 18]	No
Younger age at diagnosis (<47 years old) [5, 18]	Yes (46 years old)
Shorter duration (<5 years) before diagnosis of GADA-positive diabetes [5]	Yes
Positive anti-TPO antibodies [24]	Yes
HLA genotype susceptible to type 1 [5]	No (protective HLA genotype)
History of sulfonylureas use [25]	No

Abbreviations: ELISA, enzyme-linked immunosorbent assay; GADA, antiglutamic acid decarboxylase antibody; LADA, latent autoimmune diabetes in adults.

## Data Availability

The data supporting the findings of this study are available from the corresponding author upon reasonable request.

## References

[1] Nishimura A., Matsumura K., Kikuno S. (2019). Slowly Progressive Type 1 Diabetes Mellitus: Current Knowledge And Future Perspectives, Diabetes Metab. *Diabetes, Metabolic Syndrome and Obesity: Targets and Therapy*.

[2] Shimada A., Kawasaki E., Abiru N. (2024). New Diagnostic Criteria, 2023 for Slowly Progressive Type 1 Diabetes (SPIDDM): Report From Committee on Type 1 Diabetes of the Japan Diabetes Society (English Version). *Journal of Diabetes Investigation*.

[3] Imagawa A., Hanafusa T., Miyagawa J.-I., Matsuzawa Y. (2000). A Novel Subtype of Type 1 Diabetes Mellitus Characterized by a Rapid Onset and an Absence of Diabetes-Related Antibodies. *New England Journal of Medicine*.

[4] ElSayed N. A., Aleppo G., Aroda V. R. (2023). Classification and Diagnosis of Diabetes: Standards of Care in Diabetes—2023. *Diabetes Care*.

[5] Yasui J., Kawasaki E., Tanaka S. (2016). Clinical and Genetic Characteristics of Non-Insulin-Requiring Glutamic Acid Decarboxylase (GAD) Autoantibody-Positive Diabetes: A Nationwide Survey in Japan. *PLoS ONE*.

[6] Wada E., Onoue T., Kinoshita T. (2021). Adult-Onset Autoimmune Diabetes Identified by Glutamic Acid Decarboxylase Autoantibodies: A Retrospective Cohort Study. *Diabetologia*.

[7] Buzzetti R., Di Pietro S., Giaccari A. (2007). High Titer of Autoantibodies to GAD Identifies a Specific Phenotype of Adult-Onset Autoimmune Diabetes. *Diabetes Care*.

[8] Maruyama T., Tanaka S., Shimada A. (2008). Insulin Intervention in Slowly Progressive Insulin-Dependent (Type 1) Diabetes Mellitus. *The Journal of Clinical Endocrinology & Metabolism*.

[9] Cabrera-Rode E., Perich P., Diaz-Horta O. (2002). Slowly Progressing Type 1 Diabetes: Persistence of Islet Cell Autoantibodies is Related to Glibenclamide Treatment. *Autoimmunity*.

[10] Munakata Y., Yamada T., Takahashi K. (2012). A Case of Slowly Progressive Type 1 Diabetes With Insulin Independence Maintained for 10 Years With Alpha-Glucosidase Inhibitor Monotherapy. *Internal Medicine*.

[11] Zinman B., Kahn S. E., Haffner S. M. (2004). Phenotypic Characteristics of GAD Antibody-Positive Recently Diagnosed Patients With Type 2 Diabetes in North America and Europe. *Diabetes*.

[12] Buzzetti R., Tuomi T., Mauricio D. (2020). Management of Latent Autoimmune Diabetes in Adults: A Consensus Statement From An International Expert Panel. *Diabetes*.

[13] Rajkumar V., Levine S. N. (2022). *Latent Autoimmune Diabetes*.

[14] Howson J. M., Stevens H., Smyth D. J. (2011). Evidence that HLA Class I and II Associations With Type 1 Diabetes, Autoantibodies to GAD and Autoantibodies to IA-2, Are Distinct. *Diabetes*.

[15] Dalakas M. C. (2024). Stiff-Person Syndrome and Related Disorders—Diagnosis, Mechanisms and Therapies. *Nature Reviews Neurology*.

[16] Koopman A. D. M., Beulens J. W., Voerman E. (2019). The Association Between GAD65 Antibody Levels and Incident Type 2 Diabetes Mellitus in an Adult Population: A Meta-Analysis. *Metabolism—Clinical and Experimental*.

[17] Pieralice S., Pozzilli P. (2018). Latent Autoimmune Diabetes in Adults: A Review on Clinical Implications and Management. *Diabetes & Metabolism Journal*.

[18] Li X., Chen Y., Xie Y. (2020). Decline Pattern of Beta-Cell Function in Adult-Onset Latent Autoimmune Diabetes: An 8-Year Prospective Study. *The Journal of Clinical Endocrinology & Metabolism*.

[19] Wei Y., Herzog K., Ahlqvist E. (2023). All-Cause Mortality and Cardiovascular and Microvascular Diseases in Latent Autoimmune Diabetes in Adults. *Diabetes Care*.

[20] Kawasaki E., Shimada A., Imagawa A. (2023). Committee of Type 1 Diabetes, Japan Diabetes Society, Bivalent GAD Autoantibody ELISA Improves Clinical Utility and Risk Prediction for Adult Autoimmune Diabetes. *Journal of Diabetes Investigation*.

[21] Insel R. A., Dunne J. L., Atkinson M. A. (2015). Staging Presymptomatic Type 1 Diabetes: A Scientific Statement of JDRF, the Endocrine Society, and the American Diabetes Association. *Diabetes Care*.

[22] Kawasaki E., Nakamura K., Kuriya G. (2010). Autoantibodies to Insulin, Insulinoma-Associated Antigen-2, and Zinc transporter 8 Improve the Prediction of Early Insulin Requirement in Adult-Onset Autoimmune Diabetes. *The Journal of Clinical Endocrinology & Metabolism*.

[23] Borg H., Gottsäter A., Fernlund P., Sundkvist G. (2002). A 12-Year Prospective Study of the Relationship Between Islet Antibodies and *β*-Cell Function At and After the Diagnosis in Patients With Adult-Onset Diabetes. *Diabetes*.

[24] Murao S., Kondo S., Ohashi J. (2008). Anti-Thyroid Peroxidase Antibody, IA-2 Antibody, and Fasting C-Peptide Levels Predict Beta Cell Failure in Patients With Latent Autoimmune Diabetes in Adults (LADA)—a 5-Year Follow-up of the Ehime Study. *Diabetes Research and Clinical Practice*.

[25] Maruyama T., Shimada A., Kanatsuka A. (2003). Multicenter Prevention Trial of Slowly Progressive Type 1 Diabetes With Small Dose of Insulin (the Tokyo Study): Preliminary Report. *Annals of the New York Academy of Sciences*.

[26] Kawabata Y., Ikegami H., Kawaguchi Y. (2002). Asian-Specific HLA Haplotypes Reveal Heterogeneity of the Contribution of HLA-DR and -DQ Haplotypes to Susceptibility to Type 1 Diabetes. *Diabetes*.

[27] Awata T., Shimada A., Maruyama T. (2017). Possible Long-Term Efficacy of Sitagliptin, a Dipeptidyl Peptidase-4 Inhibitor, for Slowly Progressive Type 1 Diabetes (SPIDDM) in the Stage of Non-Insulin-Dependency: An Open-Label Randomized Controlled Pilot Trial (SPAN-S). *Diabetes Therapy*.

[28] Yang L., Liang H., Liu X. (2021). Islet Function and Insulin Sensitivity in Latent Autoimmune Diabetes in Adults Taking Sitagliptin: A Randomized Trial. *The Journal of Clinical Endocrinology & Metabolism*.

[29] Zhao Y., Yang L., Xiang Y. (2014). Dipeptidyl Peptidase 4 Inhibitor Sitagliptin Maintains *β*-Cell Function in Patients With Recent-Onset Latent Autoimmune Diabetes in Adults: One Year Prospective Study. *The Journal of Clinical Endocrinology & Metabolism*.

[30] Johansen O. E., Boehm B. O., Grill V. (2014). C-Peptide Levels in Latent Autoimmune Diabetes in Adults Treated With Linagliptin Versus Glimepiride: Exploratory Results From a 2-Year Double-Blind, Randomized, Controlled Study. *Diabetes Care*.

[31] Pozzilli P., Leslie R. D., Peters A. L. (2018). Dulaglutide Treatment Results in Effective Glycaemic Control in Latent Autoimmune Diabetes in Adults (LADA): A *post-hoc* Analysis of the AWARD-2, -4 and -5 Trials. *Diabetes, Obesity and Metabolism*.

